# Lactoferrin: A Nutraceutical with Activity against Colorectal Cancer

**DOI:** 10.3389/fphar.2022.855852

**Published:** 2022-02-21

**Authors:** Gerardo Ramírez-Rico, Maria Elisa Drago-Serrano, Nidia León-Sicairos, Mireya de la Garza

**Affiliations:** ^1^ Departamento de Biología Celular, Centro de Investigación y de Estudios Avanzados Del Instituto Politécnico Nacional (CINVESTAV-IPN), México City, Mexico; ^2^ Facultad de Estudios Superiores Cuautitlán, Universidad Nacional Autónoma de México (UNAM), México City, Mexico; ^3^ Departamento de Sistemas Biológicos, Universidad Autónoma Metropolitana Unidad Xochimilco, Mexico City, Mexico; ^4^ Centro de Investigación Aplicada a La Salud Pública (CIASaP), Facultad de Medicina, Universidad Autónoma de Sinaloa, Culiacán, Mexico; ^5^ Hospital Pediátrico de Sinaloa, Culiacán, Mexico

**Keywords:** colorectal cancer, inflammation, lactoferrin, bowel, clinical trials

## Abstract

Homeostasis in the human body results from the tight regulation of several events, since too little inflammation disrupts the process of tissue repair and remodeling, whereas too much exerts a collateral effect by causing tissue damage with life-threatening consequences. In some clinical conditions, such as inflammatory bowel disease (IBD), inflammation functions as a double-edged sword by either enabling or inhibiting cancer development and progression. Generally, cancer develops through evasion mechanisms that regulate cell growth, causing a high rate of uncontrolled proliferation, and mechanisms for evading cell death, such as apoptosis. Moreover, chronic inflammation is a factor that contributes to colorectal cancer (CRC), as observed in individuals with IBD; all these conditions favor an increased rate of angiogenesis and eventual metastasis. Lactoferrin (Lf) is a mammalian iron-binding multifunctional glycoprotein regarded as a natural compound that up- and downregulates both humoral and cellular components of immunity involved in regulating the inflammatory response and maintaining gut homeostasis. Human and bovine Lf share high sequence homology and have very similar antimicrobial, anti-inflammatory, and immunomodulatory activities. Bovine Lf from milk is considered a safe molecule and is commercially available in large quantities. This review mainly focuses on the regulatory effects of orally administered bovine Lf on the inflammatory response associated with CRC; this approach indicates that CRC is one of the most frequently diagnosed cancers and affects the intestinal tract with high clinical and epidemiologic relevance. Thus, this review may provide foundations for the potential use of bovine Lf alone or as a natural adjunct agent to increase the effectiveness and reduce the side effects of anticancer chemotherapy.

## 1 Introduction

The aim of this review is mainly to discuss the effects of bovine Lf (bLf) on colorectal cancer (CRC) and the inflammatory response associated with this disease. CRC has high morbidity and mortality rates worldwide. It is the third leading cause of cancer-related deaths, with approximately 0.7 million deaths occurring per year (Kanwar et al., 2015). CRC is a life-threatening cancer affecting the intestinal tract with clinical and epidemiological impacts (Triantafillidis et al., 2009). Some diseases are associated with a high risk of CRC, such as familial adenomatous polyposis and hereditary nonpolyposis CRC, as well as clinical variants of inflammatory bowel diseases (IBDs), mainly ulcerative colitis and Crohn’s disease (Itzkowitz and Yio, 2004; Cutone et al., 2020). CRC has a genetic background in individuals with familial adenomatous polyposis and hereditary nonpolyposis, whereas in patients with IBD, cancer is associated with chronic inflammation (Itzkowitz and Yio, 2004). Patients with IBD are also at risk of suffering small bowel adenocarcinoma and pouch and rectal neoplasms (Triantafillidis et al., 2009). CRC comprises two clinical forms: sporadic CRC and CRC associated with colitis. In sporadic CRC, adenomatous polyps (adenomas) are a major precursor. In CRC associated with colitis, several forms of epithelium dysplasia are observed: polypoid or flat, localized, diffuse or multifocal (Itzkowitz and Yio, 2004). Several factors, such as the interaction with the gut microbiota, genetic background, and immune framework, provide conditions for tumor development that are more frequently found in the colon than in the small intestine (Pancione et al., 2014).

### 1.1 Colorectal Cancer

All clinical forms of CRC arise from cumulative mutations in cancer regulatory genes or epigenetic alterations in the villi and crypts of the intestinal epithelial cell layer, leading to dysplastic lesions (Simon, 2016). Dysplastic lesions develop slowly from small benign polyps with a low grade of dysplasia and progress to precancerous large polyps with a high degree of dysplasia (low degree of cellular and structural atypia). Enlarged polyps display a greater potential to become localized adenomas that invade the gut wall, and their high rate of neovascularization enables their spread and establishment in lymphatic nodes and adjacent organs, such as the liver, resulting in metastasis with fatal outcomes (Pancione et al., 2014; Simon, 2016). Generally, CRC development results from evasion mechanisms that regulate cell growth, causing a high rate of uncontrolled proliferation. Mechanisms for evading cell death, such as apoptosis and chronic inflammation, are contributing factors to CRC, as observed in patients with IBDs; all these conditions favor an increased rate of angiogenesis and eventual metastasis (Koudougou et al., 2013; Pancione et al., 2014; Jacobs et al., 2015).

Surgery is the first treatment option for nonmetastatic CRC. Patients with advanced CRC additionally require radiotherapy and/or chemotherapy, in which drugs such as 5-fluorouracil (5-FU), oxaliplatin (OXA) (Eloxatin^®^), and irinotecan (IRI) are used. Furthermore, combined therapy is commonly used. Hand-foot syndrome due to 5-FU or neuro-, oto-, and nephrotoxicity due to OXA are the main unpleasant side effects. Furthermore, a poor response is regularly detected, mainly due to the multidrug resistance of cancer cells. Currently, monoclonal antibodies and multikinase inhibitors have been utilized to treat CRC (Cabeza et al., 2020).

### 1.2 Intestinal Inflammation and Immune Response in CRC

The development and establishment of CRC evoke the responses of humoral and cell components of host immunity that in turn also participate in the inflammatory response. The interplay between CRC and the immune response may transit through three stages: immune surveillance, equilibrium phase, and immune escape (Koudougou et al., 2013; Jacobs et al., 2015). The immune surveillance phase is accomplished by innate and adaptive immune components that collaborate to eliminate potential tumor cells, mainly if a few cells are present (Koudougou et al., 2013). Immune surveillance encompasses very complex events, including apoptosis of tumor cells, inflammation and the immune response; all these events are mediated by components with an anticancer role in CRC, including innate immune cells such as natural killer cells (NKs), M1-type macrophages, dendritic cells (DCs), mast cells and adaptive immune cells such as TCD8+ cytotoxic cells, TCD4+ helper cells (Th1 phenotype secreting interferon (IFN)-γ, IL-12, and other proteins), Treg cells and Th17 helper cells (Koudougou et al., 2013; Wallace et al., 2014; Jacobs et al., 2015; Abraha and Ketema, 2016). When immune surveillance is surpassed, the equilibrium phase is undetected in the clinic, and the role of the immune system is constrained to limit the growth of tumor cells. The last phase corresponds to immune escape, which is clinically detectable and results from the cumulative evasion of the immune response by tumor cells; this phase arises from immune editing that results from the selection of clones that evade the pressure exerted by the immune system (Koudougou et al., 2013; Pancione et al., 2014; Jacobs et al., 2015).

The intestinal mucosal compartment is the site in which a physiological inflammatory response orchestrated by innate and adaptive mechanisms mediated by intestinal epithelial cells and a wide array of immunocompetent cells in the lamina propria, such as DCs, plays a key role in maintaining gut homeostasis (Kelsall, 2008). The inflammatory response is elicited by germline-encoded pattern-recognized receptors (PRRs) expressed in several cell types that interact with their ligands, namely, pathogen-associated molecular patterns (PAMPs) or danger-associated molecular patterns (DAMPs). Some PRRs comprise a large family of receptors, such as Toll-like receptors (TLRs) (Cui et al., 2014).

Upon ligand binding, TLRs trigger signaling pathways, resulting in the activation and translocation of nuclear factor (NF)-κB to the nucleus. NF-κB modulates the expression of proinflammatory cytokines such as interleukin (IL)-1, IL-18, type I interferon (IFN-α and IFN-β), tumor necrosis factor (TNF)-α, and chemoattractant cytokines (chemokines). Another class of PRRs includes NOD-like receptors (NLRs), some of which, such as NLRP1, NLRP3 and NLRP6, function as sensors or adaptors forming “inflammasomes” (Cui et al., 2014). Activation of inflammasomes by PAMPs and/or DAMPs induces signaling pathways and the subsequent activation of caspase-1, which cleaves the inactive pro-forms of cytokines (IL-1 and IL-18) to generate their active forms. Both IL-1β and IL-18 are potent inducers of cancer cell killing by cytotoxic cells (Fabbi et al., 2015). In addition to generating active proinflammatory cytokines, some inflammasomes regulate cell death in response to microbial and endogenous danger signals (Cui et al., 2014; Parlato and Yeretssian, 2014).

Signaling pathways elicited by PRRs seem to exert a dual effect on cancer cell development. On the one hand, abnormal activation of NF-κB and inflammasomes via TLRs and NLRPs and the resulting cytokine production are the primary causes of chronic inflammation in patients suffering from IBD associated with CRC (Itzkowitz and Yio, 2004; Cui et al., 2014). Loss of control of inflammation, a high rate of cell proliferation, and a low level of apoptotic death exert deleterious effect on gut homeostasis, enabling the formation of conditions for cell transformation and malignancies in the intestine (Itzkowitz and Yio, 2004; Cui et al., 2014; Abraha and Ketema, 2016). On the other hand, PRRs exert an antitumor effect to inhibit tumor progression, showing their dual functions in cancer cells; however, the underlying mechanisms resulting in pro- and anticancer effects have not yet been completely elucidated (Cui et al., 2014). The production of proinflammatory and chemoattractant cytokines exerts a strong effect on the response of components of the innate and adaptive branches of intestinal immunity that accomplishes immune surveillance to sense and eliminate cells with potentially aberrant development (Koudougou et al., 2013; Jacobs et al., 2015). Under normal conditions, physiological inflammation is observed and results from the balance between the pro- and anti-inflammatory interleukin responses from Th1 and Th2 cells located in the intestinal lamina propria (Wallace et al., 2014). Loss of Th1/Th2 regulation may lead to chronic inflammation, which is regarded as a critical factor that favors CRC development; however, a polarized Th1 response may protect against gut dysplasia (Itzkowitz and Yio, 2004; Rizzo et al., 2011). Under conditions of chronic inflammation, IFN-γ secretion by Th1 lymphocytes seems to play a pivotal role in preventing tumor cell proliferation, while IL-13 derived from Th2 lymphocytes and TNF-α, IL-6, and IL-17 produced by Th17 cells promote dysplastic cell proliferation and tumor growth (Rizzo et al., 2011). Interestingly, milk contains bioactive components that promote the Th1 cytokine response while suppressing the Th17 and Th2 responses in the large intestinal lamina propria, as documented in immune milk-fed mice with dextran sulfate-induced colitis (Wang et al., 2014). Thus, humoral and cellular mediators of inflammation and adaptive branches of mucosal immunity seem to function as a double-edged sword in the regulation of cancer cells such that their beneficial effect may be altered by natural immunomodulators present in milk, such as lactoferrin (Lf).

### 1.3 Lactoferrin Overview

Lactoferrin is a glycoprotein of approximately 80 kDa that belongs to the family of iron transporter proteins (transferrins). Lf differs from other members of the family because it has the highest affinity for iron (Aisen and Leibman, 1972). Lf is a one high cationic polypeptide chain that is highly conserved among mammals (Drago-Serrano et al., 2017). Sorensen and Sorensen identified Lf in bovine milk for the first time in 1939, but it was isolated from human and bovine milk in 1960 (Sorensen and Sorensen, 1940; Groves, 1960; Johansson, 1960). Lf is the second most abundant protein in human milkLf also is present in low quantities in other secretions, such as saliva, bile, semen, tears, and bronchial and intestinal secretions. Lf is part of the secondary granules of neutrophils (Steijns, 2001). In human milk, the Lf concentration (2–4 mg/ml) is higher than that in milk from other species, such as bovine (0.02 and 0.2 mg/ml) or pig, mouse, and horse milk (0.2 and 2 mg/ml). Milk from rats, rabbits, and dogs contains less than 0.05 mg Lf/mL (Masson and Heremans, 1971; Conesa et al., 2008). In humans, the Lf concentration in colostrum is high (6–8 mg/ml), and it has been suggested to provide protection for babies against infectious diseases (Artym and Zimecki, 2005).

Lf is present in two different forms, depending on its iron chelation state, iron-free (apo-Lf) or bound to two Fe^3+^ iron ions (holo-Lf) (Baker and Baker, 2004). Lf exerts distinct effects on microorganisms in accordance with its iron state. In this sense, holo-Lf may serve as an iron source for some species, while apo-Lf generally causes microbiostasis since it chelates the iron necessary for the growth of pathogens in host fluids and mucous membranes. Apo-Lf also functions as a bactericide that binds and damages the bacterial membrane (Drago-Serrano, 2006). Furthermore, the N-terminus of Lf has been shown to be responsible for the bactericidal effect, since the cleavage of this region by acid-pepsin hydrolysis generates short, active antimicrobial peptides called lactoferricins (Lfcins) and a long peptide sequence called lactoferrampin (Lfampin); in fact, these peptides exert a stronger bactericidal effect than the native molecule (Haney et al., 2012; Zarzosa-Moreno et al., 2020).

**FIGURE 1:**
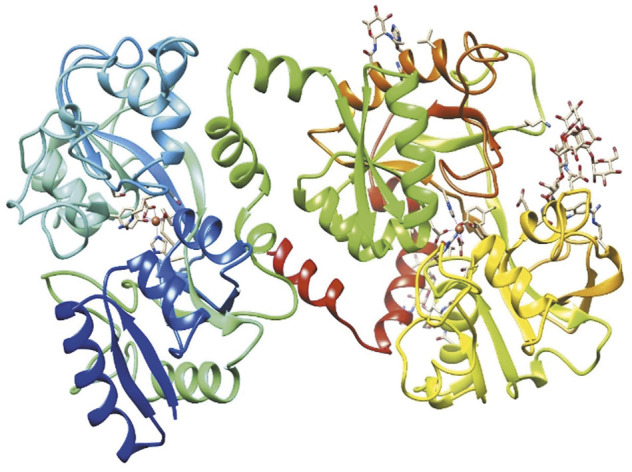
Structure of bovine diferric lactoferrin (holo-bLf). The three-dimensional structure of holo-bLf was determined by X-ray crystallography. https://www.rcsb.org/structure/1BLF. Visualized with UCSF Chimera (Pettersen et al., 2004).

Lf is considered a multifunctional protein, because several studies have reported its microbiostatic, microbicidal, anticancer and immunomodulatory properties (Tomita et al., 2009; Roseanu et al., 2010). The molecular mechanisms involve alterations in the virulence factors of microorganisms (Gomez et al., 2003; Ramírez-Rico et al., 2021); binding to host receptors or proteins (Superti et al., 2019; Chang et al., 2020); regulation of complement (Samuelsen et al., 2004); antioxidant function in oxidative stress; modulation of the cell growth and homeostasis of intestinal microbiota (Baveye et al., 1999; Vega-Bautista et al., 2019); and a positive effect on bone regeneration (Shi et al., 2020) and attenuating obesity (Brimelow et al., 2017). The tertiary structure of holo-Lf is shown in Figure 1.

## 2 Lactoferrin as an Intestinal Modulator of Immunity

Lactoferrin up- or downregulates both humoral and cellular components of immunity. The immunomodulatory effect is derived from its ability to link innate and adaptive immunity to obtain physiological equilibrium. Lf could be considered an alternative molecule for the treatment of diseases that compromise gut immune homeostasis (Legrand, 2016; Drago-Serrano et al., 2017). The balance of homeostasis results from the tight regulation of several events, since too little inflammation disrupts the process of tissue repair and remodeling, whereas too much exerts a collateral effect by causing tissue damage with life-threatening consequences (Luissint et al., 2016). In some clinical conditions, such as IBDs, inflammation functions as a double-edged sword, since it either enables or inhibits cancer development and progression (Rizzo et al., 2011; Formica et al., 2014).

### 2.1 Modulatory Effects of bLf on the Inflammatory Response in CRC

#### 2.1.1 Physiological Conditions

Bovine Lf exerts modulatory effects on mediators involved in the intestinal inflammatory response, as evidenced in healthy animals such as mice and preterm piglets (Iigo et al., 2004; Spagnuolo et al., 2007; Nguyen et al., 2016). These studies show that the modulation of mediators of intestinal inflammation by bLf depends on the duration of ingestion, dose of bLf and intestinal maturity. For example, an analysis of intestinal lymphocytes isolated from healthy female C57BL/6 mice fed pellets containing bLf for 4 days showed that bLf decreased the expression of the proinflammatory cytokine tumor necrosis factor (TNF)-α and increased the percentage and apoptosis of CD4+ cells among intestinal lymphocytes; however, the percentage and apoptosis of CD8+ cells and caspase-3 and Bcl-2 expression (encoding antiapoptotic protein) in intestinal lymphocytes were unaffected. Based on these results, the decrease in TNF-α levels induced by bLf resulted from increased apoptosis of TNF-α-producing CD4 cells without affecting CD8 cells (Spagnuolo et al., 2007). In contrast, single and/or 7 days of oral administration of bLf or bLfcin increased the activities of the proinflammatory cytokines IL-18 and IFN-γ and caspase-1 in the small intestine of healthy male BALB/c mice; these effects were related to their inhibitory effects on carcinogenesis and metastasis (Iigo et al., 2004).

Moreover, a single administration of bLf by gavage elicited the expression of NOD2-, IFN-β-, and IL-12 in cellular components of innate immunity involved in the inflammatory response in the small intestine of healthy mice (Wakabayashi et al., 2006). The dosage of bLf is critical for exerting beneficial or deleterious effects on intestinal maturation. In preterm piglets, enteral feeding formula supplemented with low doses of bLf increased the maturity and cell proliferation of intestinal cells whereas high bLf doses decreased the villous height and increased the Bax/Bcl-2 ratio (Bax is a proapoptotic protein and Bcl-2 is an antiapoptotic protein) and hypoxia inducible factor (HIF)-1, suggesting increased intestinal apoptosis and inflammation (Nguyen et al., 2016). Chronic HIF-1 expression is a risk factor for intestinal injury and CRC (Shah, 2016). These findings highlight the relevance of the bLf dosage, which provides physiological conditions of inflammatory modulation to be beneficial for gut homeostasis, as described in newborns fed milk containing Lf and other bioactive peptides, such as lysozyme, or in piglets fed recombinant human Lf (rhLf) and/or lysozyme from transgenic cattle and goats (Chatterton et al., 2013; Cooper et al., 2014). In addition, the iron saturation has a critical role on HIF-1 modulation as documented in mice treated human lactoferrin. Unlike the iron saturated counterpart, human apo-lactoferrin acts as a physiological mimetic of hypoxia stabilizes hypoxia-inducible factor-1 alpha as documented in mice (Zakharova et al., 2012).

#### 2.1.2 Inflammation

Lactoferrin exerts a dual effect on the inflammatory response in several models of intestinal cancer that seems to depend on the experimental settings. These models include mice with dextran sulfate sodium (DSS)-induced ulcerative colitis associated with cancer and Apc-Min mice as a model of spontaneous dysplasia (Kanneganti et al., 2011). As described in the murine model of DSS-induced colitis, the cytokine-inflammatory response mediated by IL-1β and/or TNF-α was decreased by hLf and bLf, although a greater beneficial effect was observed for apo-bLf than holo-bLf (Haversen et al., 2003; Li et al., 2013). These findings obtained with bLf are consistent with the anti-inflammatory effect of bovine colostrum by decreasing the expression of the proinflammatory cytokine IL-1β through NF-κB activation in HT-29 cells (An et al., 2009).

On the other hand, bLf also displays proinflammatory activity by enhancing the IL-8 response in Caco-2 cell monolayers stimulated with IFN-γ (Frioni et al., 2014). Moreover, bLf and bLfcin display proinflammatory activities by upregulating cytokines and the activity of cells involved in the inflammatory response to exert antitumor and antimetastatic effects (Wang R. et al., 2000; Shimamura et al., 2004; Kuhara et al., 2006). In mice bearing subcutaneous Co26Lu tumors with high metastatic potential, oral administration of bLf and bLfcin inhibited metastasis, accompanied by increases in the numbers of CD4+, CD8+, asialoGM1+, and IFN-γ-presenting cells and proinflammatory IL-18 and IFN-γ production in the epithelium and/or lamina propria of the small intestine and colon (Wang R. et al., 2000; Kuhara et al., 2006). Bovine Lf increased the expression of proinflammatory cytokines such as type I IFNs, i.e., IFN-α and IFN-β, in Peyer’s patches and mesenteric lymph nodes to induce the activation of cytotoxic cells involved in killing tumor cells (Kuhara et al., 2006). The anticancer effects of bLf were ascribed to its ability to induce the expression of IFN-γ and caspase-1 activation, as found in the murine small intestine. IFN-γ production induced by bLf triggered the cleavage of inactive pro-caspase-1 to generate the active form caspase-1, which in turn cleaved the inactive pro-IL18 to the active IL-18 derivative. Thus, bLf induces IL-18 production through an IFN-γ-dependent mechanism, while bLf induction of IL-18 activation depends on caspase-1 (Iigo et al., 2009). On the other hand, liposomal Lf inhibits the mRNA expression of TNF-α, a proinflammatory cytokine involved in CRC carcinogenesis that is present in human RKO and RCN-9 cells, both of which are CRC cell lines (Sugihara et al., 2017). This presentation of Lf preserves digestion in the stomach and promotes better absorption in the intestinal tract, as described below.

The extent of iron saturation and treatment duration seem to be involved in some antitumor properties of bLf. Experimental assays in tumor-bearing mice showed that unlike the apo form, only fully iron-saturated bLf functioned as an adjuvant for anticancer chemotherapy drugs by increasing Th1 (TNF-α, IFN-γ, and IL-18) and Th2 (IL-4, -5, -6, and -10) responses and nitric oxide (NO) production in homogenates from the small intestine; moreover, the protective effect required bLf feeding for 2 weeks prior to chemotherapy (Kanwar et al., 2008). Unlike Th1 cells, the role of the Th2 response in protection against tumor cells is unclear since it is associated with cancer development (Rizzo et al., 2011; Grizzi et al., 2013; Formica et al., 2014). Moreover, a role of iron in the antitumor action of bLf may be to potentiate the modulatory and effector action of immune cells, as reported in an *in vitro* study in which only iron-loaded Lf stimulated the proliferation and differentiation of cultured lymphoblastic T cells (Bi et al., 1997). Under healthy conditions, iron-unsaturated bLf administered by intragastric intubation or orally also elicited both Th1 (IFN-γ) and Th2 (IL-10) responses in mesenteric nodules and the distal small intestine (Takakura et al., 2006; Arciniega-Martinez et al., 2016) or only a Th1 response, as observed in Peyer’s patches isolated from the whole length of the mouse small intestine (Sfeir et al., 2004). Although the role of iron in the antitumor actions of bLf has not been completely elucidated, the reversible iron-binding properties of bLf may be beneficial to balance the iron levels involved in the generation of reactive oxygen species (ROS), causing tissue damage (Kruzel et al., 2007). The modulatory effect of bLf on ROS generation has suggested to protect against inflammation-associated CRC (Fujita et al., 2002; Burrow et al., 2011; Li et al., 2013). Similarly, Burlaka reported that the concentration of Lf varied according to the type of differentiation of neoplasia during CRC metastasis. Lf levels are higher in poorly differentiated neoplasms than in well-differentiated tumors. These results correlated with the iron content in these types of neoplasms, where the iron concentration is four times higher than the value in intestinal mucosa without pathology (Burlaka et al., 2019). Indeed, ROS production during chronic inflammation is associated with the oxidation of DNA, resulting in neoplastic transformation (Itzkowitz and Yio, 2004).

**FIGURE 2:**
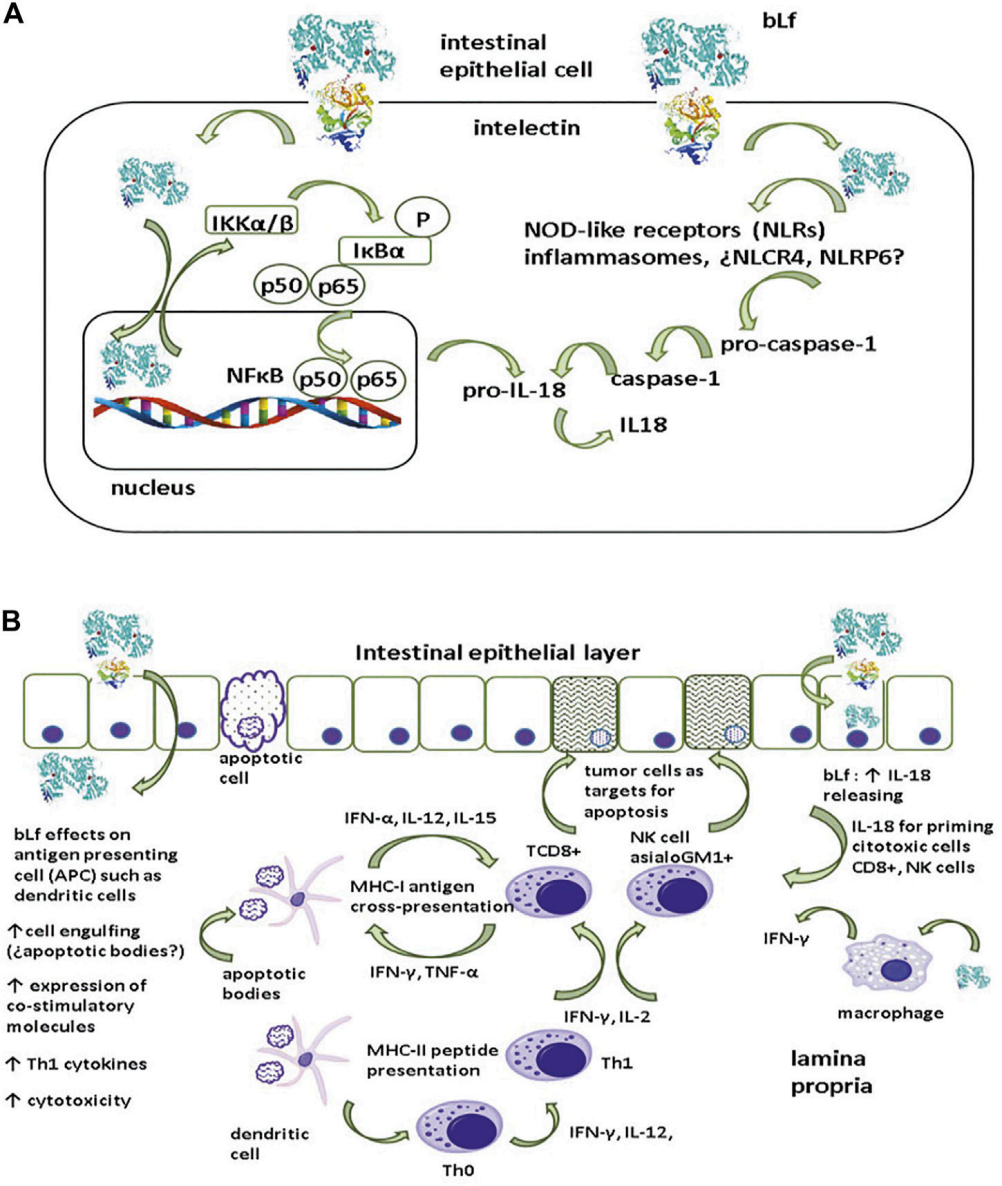
Presumable mechanism of pro-inflammatory cytokine generation by lactoferrin. **(A)** After being endocytosed by intelectin (INTL) by epithelial cells, lactoferrin (Lf) is targeted to nucleus where induces the upmodulation of IKKα/β expression. Once activated, IKK α/β undertakes the phosphorylation and the concomitant degradation of IκBα resulting in the release of NFκB (p50/p65) to be translocated to nucleus to upmodulate the expression of pro-IL18. Additionally, translocation of bLf to cytosol may elicit the activation of Nod-like receptors inflammasomes (¿NLCR4, NLRP6?) involved in the conversion of pro-caspase 1 into the active caspase 1. Caspase 1 accomplishes the conversion of the inactive pro-IL18 into active IL-18. **(B)** After being endocytosed via INTL, Lf induces the production of IL-18 with a pivotal role in the activation of cytotoxic cells essential for the apoptosis of tumor cells. Additionally, INTL receptor may enable the translocation of Lf to lamina propria, where Lf may display a wide array of modulatory actions on macrophages and dendritic cells (DCs). Macrophages are important source of IL-18 and DCs have an essential role as antigen presenting cells that orchestrate the activation of cytotoxic cells and the polarization of pro-inflammatory Th1 response intended for the elimination of tumoral cells via apoptosis (see text for details).

Mechanisms underlying the anticancer activity of bLf are difficult to recapitulate since the multistep character entails the interaction of the immune system with cancer cells, as well as the wide array of bLf properties that modulate innate and adaptive immunity and subsequent inflammation. An overall mechanism of inflammation and its role in dysplastic epithelial cells (Figure 2A) entails bLf uptake and translocation through the epithelial layer via receptors, as observed in Caco-2 monolayers (Lonnerdal et al., 2011). After being endocytosed via the intelectin (ITLN-1) receptor, Lf is targeted to the nucleus, where it upregulates IKKα/β expression (Oh et al., 2004; Suzuki et al., 2008). ITLN is a glycoprotein of 105 kDa expressed at the apical membrane of intestinal epithelial cells (Suzuki et al., 2008). Once activated, IKK α/β phosphorylates and concomitantly degrades IκBα, resulting in the release of NFκB (p50/p65) and its translocation to the nucleus to upregulate the expression of the proinflammatory cytokines IL-1β and IL18 (Cui et al., 2014). Although the modulatory effect of bLf on inflammasomes is not known, it is known that bLf translocates to the cytosol where it may activate NOD-like receptor inflammasomes expressed in the intestine (NLCR4 and NLRP6) to regulate the conversion of pro-caspase 1 into active caspase 1 that subsequently cleaves pro-IL18 into the active form IL-18 (Cui et al., 2014).

Receptor-mediated endocytosis of bLf may drive some outcomes (Figure 2B). After being endocytosed via the ITLN-1 receptor within epithelial cells, Lf may induce IL-18 production by epithelial cells, which has a pivotal role in the activation of the cytotoxic activity of effector NK and TCD8+ cells (Wang R. et al., 2000; Kuhara et al., 2000). Additionally, the ITLN-1 receptor may facilitate the translocation of Lf to the lamina propria*,* where it exerts a wide array of modulatory effects; for example, bLf induces macrophage production of IL-18, which is regarded as an anticancer cytokine due to its Th1 polarization activity (Iigo et al., 2009; Fabbi et al., 2015). Lf regulates DCs, which have an essential role as antigen-presenting cells (Puddu et al., 2009). DCs modulate the generation of IL-12 and IFN-γ by Th0 cells to polarize the proinflammatory Th1 response designed to eliminate tumor cells (Kelsall, 2008). Moreover, DCs accomplish cross-presentation through the MHC-I restricted presentation of peptides derived from antigens released by apoptotic cells (for example, epithelial cells) to TCD8+ cytotoxic lymphocytes (Spel et al., 2013). Under chronic inflammatory conditions, IFN-γ secretion by Th1 lymphocytes seems to play a pivotal role in the prevention of tumor cell proliferation (Rizzo et al., 2011); thus, polarization toward a Th1 response by bLf may protect against colon dysplasia.

#### 2.1.3 Cancer

Bovine Lf has been proven to be a prophylactic and chemotherapeutic agent in practically all stages of intestinal cancer including cell proliferation, angiogenesis and metastasis by modulating the innate and adaptive immune responses. Anticancer effects of bLf include its ability: 1) to elicit IL-18 and caspase-1 responses 2) to induce the activation of natural killer cells (NKs), CD4+, CD8+, and IFN-γ T cells, 3) to induce apoptosis of carcinogenic epithelial cells in colon via Fas, caspase-3 and caspase-8 activation and 4) to inhibit angiogenesis (Tsuda et al., 2010). bLf and even its pepsin derivative, hydrolysate bLfcin, have been tested as chemopreventive, anti-metastatic and anti-tumoral agents in the small and large intestine in experimental assays conducted in rats and mice. These include spontaneous polyp development throughout the intestine in adenomatous polyposis coli (Apc) multiple intestinal neoplasia (Min) mouse (Ushida et al., 1998; Ushida et al., 1999), subcutaneous implantation of highly metastatic colon carcinoma cells in BALB/c mice (Iigo et al., 1999) and azoxymethane (AOM) induced colon tumor in F344 rats (Sekine et al., 1997; Tsuda et al., 1998; Fujita et al., 2002; Ye et al., 2014) (Table 1).

**TABLE 1:** Trials in humans and assays in animal models with lactoferrin.

Reference	Lactoferrin origin/dose/duration	Type of tumor/drug	Species	Effect
Studies in animal models				
Sekine et al. (1997)	bLf (0.2 or 2% body weight) 36 weeks	Adenocarcinoma in LI induced by AOM	F344 rats	Significantly reduced with both doses
Tsuda et al. (1998)	bLf (15 mg/kg) 4 or 13 weeks	Adenocarcinoma in LI induced by AOM	F344 rats	Decreased numbers of ACF
	bLf (2, 0.2%, hydrolysate, or 0.1% Lfcin) 36 weeks	Colon adenocarcinomas induced by AOM	F344 rats	Decreased incidences of neoplasia, ACF and β-glucuronidase activity
Ushida et al. (1998)	bLf (0.2 or 2% body weight) 8 weeks	Familial adenomatous polyposis and sporadic colon	ApcMin mouse	Reduction and significant suppression of polyps
	bLf (0.2 or 2% body weight) 8 weeks	CRC induced by AOM	F344 rats	Inhibition of development of ACF
Iigo et al. (1999)	bLf hydrolysate, Lfcin (30, 100, 300 mg/kg) 3–9 or 3–23 days	Implants of the highly metastatic colon carcinoma 26 cells	BALB/c mice	Inhibition of metastasis
Ushida et al. (1999)	bLf (2, 0.2, 0.02 or 0.002% body weight) 8 weeks	Multi-organ carcinogenesis model induced by DEN, DHPN, NMBA	F344 rats	Reduction, suppression and decrease of neoplastic lesions in different organs
Kuhara et al. (2000)	bLf hydrolysate (100 or 300 mg/kg/day) 1 week	Co26Lu cells were Injected	BALB/c mice	Significant inhibitory effect on metastasis, before and after tumor implantation
		Colon carcinoma on lungs		
Iigo et al. (2009)	bLf hydrolysate, Lfcin, Tf, (30 or 300 mg/kg/day) 7 or 22 days	Co26Lu cells were injected	GKO or BALB/c mice	Inhibition of tumor growth and metastasis
		Colon carcinoma on lungs		
Ye et al. (2014)	18 weeks	CAC induced by AOM and DSS	Lactoferrin knockout mice	Lf was the key in colorectal mucosal immunity and inflammation
Tanaka et al. (2021)	bLf (2% body weight)83 days	Cancer colon induced by AOM and DSS	C57BL/6 J mice	Few lesions in the colon and less weight loss
Clinical trials in humans				
Kozu et al. (2009)	bLf (1.5 g or 3.0 g daily) 12 months	Polyps (adenomas)	Humans (Age 40–75)	Significantly retarded adenomatous polyp growth
Iigo et al. (2014)	bLf (1.5 g or 3.0 g daily) 1 year	Adenomatous colorectal polyps	Humans (Age 63 or younger)	Suppressed growth of colorectal polyps
Moastafa et al. (2014)	bLf (250 mg/day) 3 months	Colorectal cancer	Humans (Age 20–71	Clinically beneficial effect to colorectal cancer patients with better disease prognosis

ACF, aberrant crypt foci; AOM, azoxymethane; CRC, colorectal cancer; DEN, diethylnitrosamine; DHPN, dihydroxy-di-N-propylnitrosamine; NMBA, N-nitrosomethylbenzylamine; DSS, dextran sulfate sodium; CAC, colitis-associated colon cancer.

In ApcMin mouse model, bLf inhibited the polyposis in the small intestine by decreasing the number of polyps and suppressed polyp generation without displaying toxic effects (Ushida et al., 1998). In BALB/c mice bearing subcutaneous implanted tumors of C26 mouse colon carcinoma (Co 26Lu highly metastatic in lung) both bLf and bLfcin inhibited the lung metastatic colony formation but effects on implanted tumor growth were not seen. In addition, bLf increased the number of asialoGM1+ (natural killer glycolipid marker) and CD8+ cells from peripheral blood. Interestingly, bLf did not exert anti-metastatic activity in athymic nude mice bearing Co 26Lu suggesting that asialoGM1+ and CD8+cells are involved in the anti-metastatic activity of bLf and bLfcin (Iigo et al., 1999). In AOM-induced colon tumors in male F344 rats, bLf and bLfcin had no toxic effects and inhibited the colon tumor and aberrant crypt foci by enhancing the NK cell activity essential for the elimination of tumor cells (Tsuda et al., 1998). In AOM-induced colon tumors in rats was documented that bLf had chemopreventive activity by its apoptotic ability on tumor cells resulting from the increased Fas expression as well the activation of caspase-8 and -3 (Fujita et al., 2004). Most biological functions of bLf including its role on apoptosis may result in part, to the uptake of bLf via receptor by intestinal epithelial cells and its translocation to nucleus as found with human Lf in Caco-2 cell culture assays (Suzuki et al., 2008) (Table 1).

**FIGURE 3:**
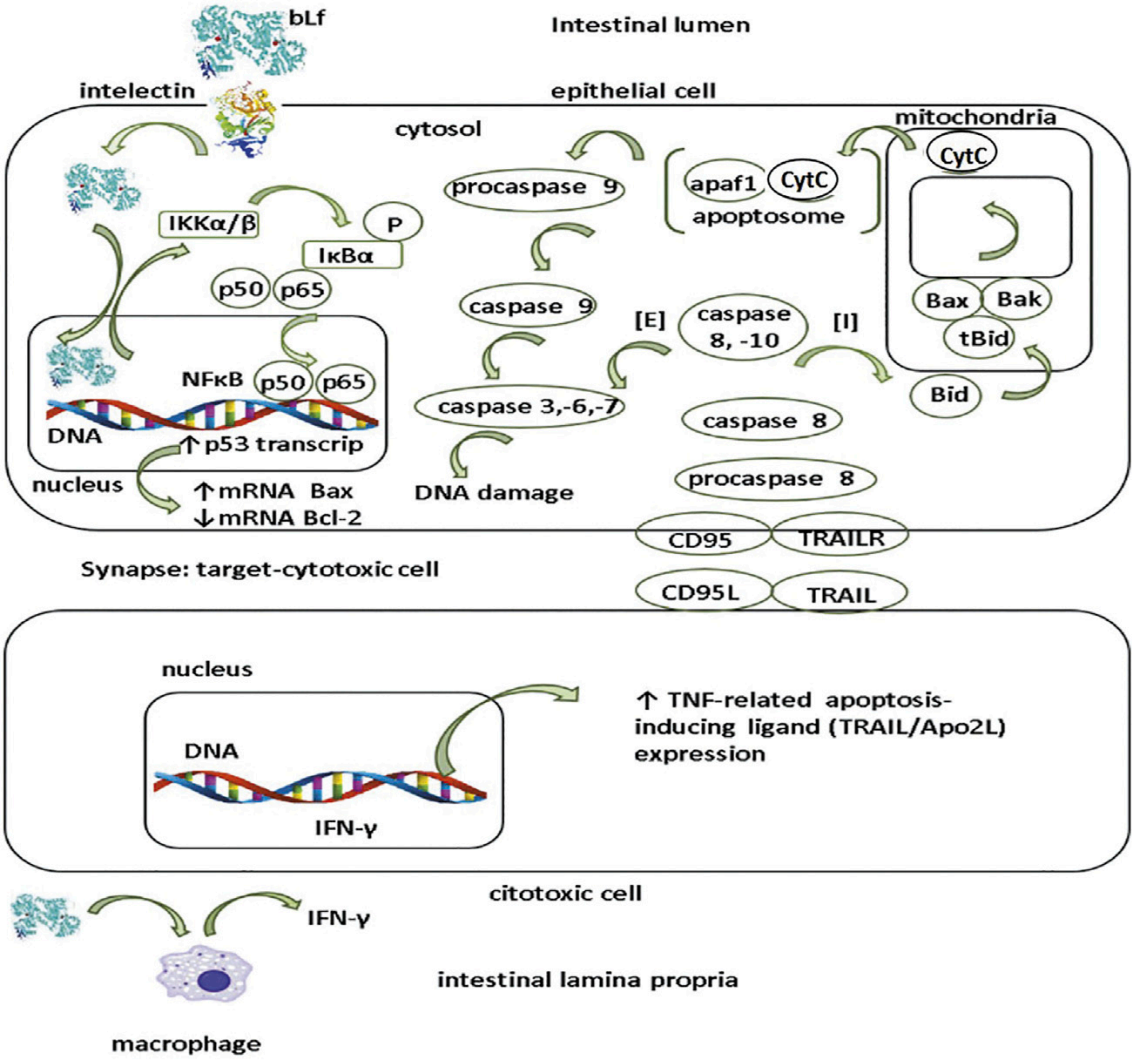
Presumable mechanism of apoptosis by lactoferrin in intestinal tumor cells. Apoptosis pathway may be elicited by lactoferrin (Lf) after being internalized by intelectin (INTL) receptor expressed by intestinal epithelial cells. After translocation, Lf is targeted to nucleus where functions as *trans*-activator of p53 promotor via NFκB promotor activation. Lf induces the IKKα/β activation and concomitant phosphorylation and degradation of IκBα resulting in the release of NFκB (p50/p65) to nucleus. Activation of p53 gene induces the expression of pro-apoptotic proteins (Bax,Bak) while decreases the expression of anti-apoptotic proteins (Bcl-2). Extrinsic (E) apoptosis pathway relies on the ligation of surface molecules on target cells CD95 (Fas/Apo1) and/or TRAILR with their corresponding ligands on the cytotoxic cells CD95L and/or TRAIL respectively. Interaction ligand-receptor enables the conversion of inactive procaspase 8 in the active form as caspase 8. Caspase 8 forms a complex with caspase 10 that triggers sequentially the cascade of activation of caspase -3,-6 and -7 resulting in DNA fragmentation. Intrinsic (I) pathway collaborates in the apoptosis of tumor cells. In this route, caspase 8 and -10 split Bid into the active form tBid (truncated Bid) which in turn activates Bax and Bak. Both Bax and Bak facilitate the outcome of cytochrome C (cytC) from the mitochondrial intermembrane space to the cytosol. Once translocated to cytosol, cytC together with apaf1, form a protein complex called as apoptosome that activates procaspase 9 into caspase 9 that in turn elicits the activation of caspase -3, -6 and -7 resulting in DNA damage. Additionally, at subepithelial level (lamina propria), Lf may enhance apoptosis of tumor cells by eliciting the IFN-γ in macrophages that upmodulates the expression of TRAIL in cytotoxic cells.

Presumable mechanism of apoptosis in tumor epithelial cells (Figure 3) involves the interaction of bLf with the ITLN receptor, and once internalized, bLf may be translocated to nucleus where acts as *trans*-activator of p53 promotor via NF-κB activation as described for human neutrophil Lf in HeLa cells (Oh et al., 2004). Lf enhances the activation of IKKα/β kinase resulting in the phosphorylation and degradation of IκBα subunit (Oh et al., 2004); the latter, leads to the release and concomitant activation of NFκB and its translocation to nucleus where induces the transcription of p53 gene in response to DNA damage may lead in cell death (Fulda and Debatin, 2006; Cui et al., 2014). p53 gene protein product induces the expression of pro-apoptotic proteins like Bax that along with Bak are pore forming proteins at mitochondrial outer membrane that permit the outcome of cytochrome C from the interspace membrane to cytosol (Fulda and Debatin, 2006; Chan and Housseau, 2008). In addition, p53 protein attenuates the expression of anti-apoptotic Bcl-2 protein in stem cells located at base of crypts in colon but absent in the murine small intestine; the role of Bcl-2 in attenuation of apoptosis in stem cells may lead to neoplastic transformation frequently found in the large than small intestine (Merritt et al., 1995).

The role of bLf on extrinsic and intrinsic apoptosis pathways is unknown but seem to underlie its anti-tumoral effects as supported in several studies (Tsuda et al., 1998; Fujita et al., 2004). Assays in Lf-knockout mice treated with AOM showed that these animals were highly prone to AOM inflammation-induced colorectal dysplasia that in turn was associated with decreased NF-κB factor signaling and modulation of apoptosis and cell growing (Ye et al., 2014). Fas (also known as CD95 or APO-1) is a transmembrane protein which belongs to the TNF receptor family endowed with conserved death domains in the intracellular region. After interacting with the ligands (Fas ligand (FasL) and TNF) the conserved death domains of Fas recruit adaptor molecules that elicit signal pathways resulting in the activation the caspase-8 and caspase-3 which are intracellular signaling components from Fas leading DNA fragmentation and ultimately death cell (Fulda and Debatin, 2006; Chan and Housseau, 2008).

Extrinsic apoptosis pathway relies on the ligation of surface molecules on target (epithelial) cells CD95 (Fas/Apo1) and/or TRAILR with their corresponding ligands on the cytotoxic (NK and TCD8+) cells CD95L and/or TRAIL respectively (Figure 3). Interaction ligand-receptor enables the conversion of inactive procaspase 8 in the active form as caspase 8. Caspase 8 forms a complex with caspase 10 that triggers sequentially the cascade of activation of caspase -3, -6 and -7 resulting in DNA fragmentation (Figure 3). Along with the extrinsic via, the intrinsic (I) pathway collaborates in the apoptosis of tumor cells. In this route, caspase 8 and -10 split Bid into the active form tBid (truncated Bid) which in turn activates Bax and Bak. Both Bax and Bak facilitate the outcome of cytochrome C (cytC) from the mitochondrial intermembrane space to the cytosol (Figure 3). Once translocated to cytosol, cytC together with apaf1, form a protein complex called as apoptosome that activates procaspase 9 into caspase 9 that in turn elicits the activation of caspase -3, -6 and -7 resulting in DNA damage and cell death (Fulda and Debatin, 2006; Chan and Housseau, 2008). As depicted in Figure 2, one presumable role of bLf after being translocated at subepithelial level is the elicitation of IFN-γ by macrophages (Iigo et al., 2009). Along with IL-18, IFN-γ contributes in priming of cytotoxic cells for killing tumor cells and in antiangiogenic mechanisms (Fabbi et al., 2015).

## 3 Lactoferrin and Its Derived Peptides in the Prevention and Therapy of CRC

Chemoprevention of cancer using natural and/or synthetic compounds is an approach to reverse, suppress, or delay the appearance of malignant cells (Zamarin and Postow, 2015; Abraha and Ketema, 2016). The development of targeted therapy has modified the treatment of CRC using agents such as bevacizumab and cetuximab that provide opportunities to treat both locally advanced and metastatic CRC. Nonetheless, all drugs used against cancer have limitations; consequently, scientists are engaged in a continuous effort to develop new therapies. Among the natural compounds, bLf exhibits a wide array of prophylactic and therapeutic effects on intestinal cancer cells, as evidenced in the animal models and human trials (Tsuda et al., 2010) described outlined below.

### 3.1 Bovine Lf Alone or as an Adjunct Agent as a Treatment for CRC: Assays in Animals

Regarding the experimental use of Lf in CRC models, Dr. Tsuda has been a pioneer in this research field since the 1990s. Sekine et al. examined azoxymethane (AOM)-induced tumors in F344 rats, and afterward, bLf was administered via injections (2 or 0.2% body weight) for 36 weeks. The incidence and number of adenocarcinomas in the large intestine were significantly reduced by both doses of bLf compared with the control group, and no toxic effects were noted (Sekine et al., 1997). In 1998, the same group of researchers documented the effect of Lf on this model, but other variables were introduced, such as other bLf-related products, doses and times of treatment. They divided the study into three experiments: in Experiments I and II, they administered bLf for 4 weeks or 13 weeks, respectively, along with two s. c. 15 mg/kg injections of AOM on days 1 and 8. The numbers of aberrant crypt foci (ACFs) were decreased by both treatments. In Experiment III, animals were administered three weekly injections of AOM and then separated into four groups that received 2 or 0.2% bLf, 2% bLf hydrolysate, or 0.1% bovine lactoferricin (bLfcin) for 36 weeks. Again, no toxic effects were noted, and the incidences of colon adenocarcinomas in the groups were 15, 25, 26.3, and 10%, respectively, in contrast to 57.5% in the control group. The number of ACFs was reduced and beta-glucuronidase activity was decreased in the cecal content of animals receiving bLf (Tsuda et al., 1998). β-Glucuronidase is a lysosomal glycosidase enzyme that catalyzes the degradation of the extracellular matrix of cancer and normal cells and the glycosaminoglycans of the cell membrane, which is important for cancer cell proliferation, invasion, and metastasis. In CRC and other types of cancer, the level of β-glucuronidase activity is increased (Anouar et al., 2019). In addition, the increase NK cell activity induced by bLf indicated that its inhibitory effect might have been related to increased cytotoxicity of immune cells (Tsuda et al., 1998). The results of this group of studies reveal that Lf and its peptides are good tools for CRC treatment and could be used successfully in patients.

Then, another model was established to determine the involvement of bLf in spontaneous intestinal polyp development. This model was assessed in mice that develop both familial adenomatous polyposis and sporadic colon cancers. A reduction in the total number of polyps in the small intestine was observed in the bLf-treated animals, along with significant suppression in the jejunum of animals receiving the 2% dose compared with untreated animals. In addition, body growth suppression (due to anemia and/or intussusception of polyps in the intestine) was alleviated, and no toxic effects were observed on the intestinal epithelium. In these experiments, animals were orally administered 0.2 or 2% bLf as a basal diet for 8 weeks (Ushida et al., 1998), similar to the model of male F344 rats that were induced to develop CRC with AOM, to determine the inhibition of the initiation and early-stage development of ACF by bLf. Although the results were not as obvious as those detected in the rat model, the data suggest that bLf may be a chemopreventive agent for intestinal polyposis.

Subsequently, the effects of bLf and the related compounds bLf hydrolysate and bLfcin on tumor growth and metastasis to the lungs were investigated in BALB/c mice bearing s.c. implants of highly metastatic colon carcinoma 26 (Co 26Lu) cells. Animals that were orally administered bLf and the bLf hydrolysate showed significant inhibition of lung metastatic colony formation from implanted tumors without appreciable effects on tumor growth, while bLfcin showed a tendency to inhibit lung metastasis. Nonetheless, in athymic nude mice bearing Co 26Lu tumors, bLf did not exhibit substantial antimetastatic activity; however, it inhibited lung metastatic colony formation, which was associated with an increase in the numbers of AsialoGM1+ and CD8+ cells in the blood. These results are important for the inhibitory effects of bLf on tumor growth and metastasis (Iigo et al., 1999). Using a rat multiorgan carcinogenesis model, the ability of bLf to inhibit tumor metastasis was tested. The model was developed in male F344 rats receiving i.p. injections of diethylnitrosamine (DEN) and dihydroxy-di-N-propylnitrosamine (DHPN) in drinking water and s.c. injections of N-nitrosomethylbenzylamine (NMBA) during the first 8 weeks (DDN treatment). Then, rats were treated with 2, 0.2, 0.02 or 0.002% bLf administered in the basal diet. Histopathological examinations of neoplastic lesions in the main organs, such as the esophagus, showed a tendency toward a reduction in the bLf-treated animals, along with a significant suppression of relatively large-sized papillomas (more than 50 mm^3^ vol) by the 0.2% dose (11% of the control). The multiplicity of adenomas and carcinomas in the lungs was also decreased in animals treated with bLf, which exerted chemopreventive effects on the esophagus and lung, in addition to the colon (Ushida et al., 1999).

In another animal model, oral administration of bLf and its hydrolysate at doses of 100 or 300 mg/kg/day for 1 week exerted a significant inhibitory effect on metastasis before and after tumor implantation (colon carcinoma of the lungs). Some specific effectors of cellular immunity were analyzed, and animals treated with bLf and the hydrolysate exhibited increased numbers of CD4+, CD8+, and asialoGM1+ cells in the spleen and peripheral blood, as well as cytotoxic activities against Yac-1 and Co 26 carcinoma. In the small intestinal epithelium, numbers of CD4+ and CD8+ cells and the production of interleukin-18 (IL-18) were markedly increased. Therefore, the inhibition of metastasis by oral administration of bLf and its pepsin hydrolysate is mediated by increasing IL-18 production in the intestinal epithelium and activating cellular immunity (Kuhara et al., 2000). Increased production of IL-18 and IFN-γ and caspase-1 activation induced by treatment with bLf are important factors contributing to the increase in intestinal mucosal immunity in tumor-bearing mice (Wang W. P. et al., 2000). In addition, bLf increased Fas expression and apoptosis in the colon mucosa of AOM-treated rats, as well as the expression of proapoptotic Bcl-2 family members (Fujita et al., 2004). Thus, bLf activated an effector pathway mediated by IFN-γ, caspase-1, and IL-18. Additionally, ingested bLf activates multiple effector pathways. For example, in GKO mice, while bLF administration did not activate the IFN-γ/caspase-1/IL-18 effector pathway, it inhibited tumor growth and metastasis by activating the IFN-alpha/IL-7 effector pathway (Iigo et al., 2009).

Ye et al. used an Lf knockout mouse model in which the mice are fertile, develop normally, and display no gross morphological abnormalities, and then chemically induced intestinal inflammation in these animals to investigate the roles of Lf in inflammation and cancer development. These mice displayed a greater susceptibility to inflammation-induced colorectal dysplasia, and this characteristic may be related to the inhibition of NF-kB and AKT/mTOR signaling, as well as the regulation of cell apoptosis and proliferation. The protective roles of Lf in colorectal mucosal immunity and inflammation-related malignant transformation, along with a deficiency in some components of the innate immune system, may lead to serious consequences under condition of chronic inflammation (Ye et al., 2014). A very interesting study was performed by Jiang and Lönnerdal using a transcriptome analysis, which indicated that bLf, cyclic LfcinB, and linear LfcinB exerted antitumor activities by differentially activating diverse signaling pathways, including p53, apoptosis, and angiopoietin signaling. *In vitro* studies using human CRC cells (HT-29) confirmed that both bLf and LfcinBs increase the expression of caspase-8, p53, and p21, critical proteins involved in tumor suppression, providing valuable information on the potential clinical applications of bLf and LfcinB in CRC therapy (Jiang and Lonnerdal, 2017).

Recently, a patient with Crohn’s disease was reported to have remained in remission for over 7 years while ingesting 1 g of bLf daily. In a placebo-controlled trial, ingestion of bLf inhibited the growth of intestinal polyps. Thus, the effects of bLf were investigated in a model of CRC related to IBD. The mice were divided into four groups: no treatment, treated with bLf, treated with AOM plus DSS, and treated with AOM + DSS + bLf. AOM was used to initiate intestinal cancer, and DSS was used to induce IBD-like inflammation in the intestine of C57BL/6 mice. The animals treated with AOM + DSS + bLF exhibited a better fecal score, fewer lesions in the colon and less weight loss than the mice treated without bLf, but no differences in the tumor burden were observed (Tanaka et al., 2021).

### 3.2 Administration of Bovine Lf Alone or as an Adjunct Agent in the Treatment of CRC: Clinical Trials

Since Lf is a natural product from the mammalian innate immune system and has been proven to lack toxicity, it has been used in clinical trials involving patients suffering from distinct types of cancer. These trials are always double-blinded, randomized and controlled. Kozu et al. conducted a clinical trial enrolling 104 patients aged 40–75 years with polyps ≤5 mm in diameter (likely to be adenomas). Participants were assigned to receive placebo, 1.5 g, or 3.0 g of bLf daily for 12 months. Adenomatous polyps were monitored using colonoscopy. In patients who ingested 3.0 g of bLf, significantly retarded adenomatous polyp growth was observed, mainly in patients aged 63 years or younger. This result was very promising because the removal of adenomatous colorectal polyps is performed as a preventive measure against CRC development; however, polyps can be ignored, and when detected, polypectomy is not always an option to eradicate a polyp. Therefore, this clinical trial suggests that daily intake of bLf might be a clinically beneficial adjunct treatment to colorectal polyp extraction (Kozu et al., 2009). Additionally, in a clinical trial published by the same group of researchers, participants who ingested bLf presented increased serum hLf levels, a possible increase in systemic NK cell activity, and increased numbers of CD4+ and CD161+ cells in the polyps. Taken together, these data suggest that bLf suppresses colorectal polyps by enhancing immune responsiveness, consistent with studies performed in rat and mouse models (Iigo et al., 2014). A clinical trial (ClinicalTrials.gov Identifier: NCT01596634) is being conducted to test whether oral bLf reduces taste disturbances in patients with CRC receiving OXA-based chemotherapy.

Another clinical trial examined two groups of patients with CRC to study the therapeutic benefit of orally administered bLf to people who received 5-FU and leucovorin calcium. The test group orally received 250 mg/day bLf in addition to chemotherapy for 3 months, whereas the control group received chemotherapy alone. Although a significant effect was observed that indicated an improvement in the mean percent change in all parameters 3 months after treatment (serum Lf level, serum glutathione-S-transferase enzyme (GST) activity, IFN-γ level, tumor marker carcinoembryonic antigen (CEA), renal and hepatic function tests, and complete blood counts), no significant difference was observed between the results from patients in the test group and in the control group (Moastafa et al., 2014). The purity and quality of bLf administered, doses, times of treatments, and, in general, an overview of the CRC state in participants must be considered.

Because several animal models of all stages of colon carcinogenesis showed promising effects and because those experiments indicated that oral administration of bLf exerts anticarcinogenic effects on the colon and other organs, the participation of bLf in inhibiting the growth of adenomatous colorectal polyps in human patients was corroborated in two clinical trials. As a nutraceutical, bLf and related products inhibit CRC through the following steps: to stop, control, or suppress processes that permit colon cancer growth; to make cancer cells more recognizable and therefore more susceptible to destruction by the immune system; enhance the killing power of immune system cells, such as T cells, natural killer cells, and macrophages; and to block or reverse the processes that change a polyp to adenocarcinoma. bLf might enhance the ability of the body to repair or replace normal cells that are damaged or destroyed by other forms of cancer treatment, such as chemotherapy or radiation; and to prevent cancer cells from spreading to other parts of the body and inhibit angiogenesis (Table 1).

## 4 Different Formulations of Lactoferrin With Potential Activity Against Colorectal Cancer

Practically, all anticancer drugs are toxic and lack selectivity since they do not distinguish between cancer and normal tissues, inducing severe systemic cytotoxicity and suppression of the immune system; these problems limit their use as treatments for many clinical conditions (Cutone et al., 2020). As described above, Lf is a natural anticancer glycoprotein from the mammalian immune innate system; in addition, Lf is an immunomodulator, and the expression of LfRs, which are expressed in most cells, is substantially increased in tumor cells (Kondapi, 2020). Lf can be used alone or in combination with anticancer drugs, resulting in an increase in drug efficacy. However, Lf is susceptible to degradation by pepsin in the stomach and by trypsin in the small intestine (Rastogi et al., 2014).

Researchers initially proposed linking or coating Lf with some product to protect it from degradation. For example, Ebrahim et al. purified a biomacromolecular complex with a high molecular weight from bovine colostrum whey that contained Lf (HMW-bLf, 250 kDa). This complex showed higher thermal stability and better resistance to gut enzyme digestion than other forms of bLf monomers. In addition, HMW-bLf displayed strong anticancer properties in terms of cytotoxicity and the inhibition of cell proliferation (Ebrahim et al., 2014). Afterward, numerous advances in the development of Lf nanoparticles (NPs) to deliver this glycoprotein with an intact structure and conformational activity, either alone or together with other drugs, using different methods have been reported, all of which use Lf as the basic product, due to its properties of dissolution, pI, and binding to diverse materials. Takeuchi et al. reported that the enteric coating of bLf nanoformulations increased drug transport to the lymphatic fluid within 3 h after intragastric administration, in addition to protecting against digestion by gastric enzymes (Takeuchi et al., 2006). The utilization of NPs containing Lf against diverse types of cancer cells has been recently reviewed (Kondapi, 2020). To the best of our knowledge, clinical trials with NPs containing Lf coupled to drugs have not been performed in patients suffering CRC. Next, we describe some examples of the use of Lf NPs in experiments with CRC cells and animal models. For an explanation of the methods used to prepare nanoparticles, diverse references must be reviewed elsewhere.

As mentioned above, OXA and 5-FU are two of the main chemotherapeutic drugs used to combat CRC in patients. However, they are toxic to the liver, kidneys and bone marrow. In 2018, Ahmed et al. evaluated the antiproliferative potential of 5-FU/OXA loaded in Lf NPs (lacto-nano-5FU and lacto-nano-oxalo) prepared using the Sol-oil method against the COLO-205 cancer cell line. In addition, the authors also i.v. injected these NPs in Wistar rats, and pharmacokinetic parameters and safety were measured. In addition to Lf NPs containing 5-FU or OXA, DSS was also administered to rats in the drinking water to accelerate the process of AOM-ACF induction. The results clearly showed that the 5-FU- and OXA-loaded NPs exhibited enhanced antiproliferative activity and a lower IC50 in the cells than the free soluble drugs. Furthermore, the NPs gently deliver drugs into colon cancer cells and remain there for a longer time than the soluble drugs. Interestingly, colon histopathology showed that the number of ACF was reduced or became normal after treatment with the nanoformulation compared to the positive controls of free drugs. On the other hand, Lf NPs improved the pharmacokinetic profile and biodistribution of the drugs in healthy rats and showed efficacy and safety in the liver and kidneys, as determined by measuring biochemical parameters, and did not reduce the blood cell count in rats with induced colon cancer.

### 4.1 Nanoliposomes Containing Lf

Nanoliposomes (NLs) are spherical vesicles whose membranes are composed mainly of one or more bilayers of a phospholipid, such as phosphatidylcholine (PC). NLs have been designed since the 1980s to increase the stability of the encapsulated material and protect it from the surrounding milieu (Chen et al., 2012). In addition, other lipids and polyethylene glycol (PEG) have been used instead of PC. NLs are ideal candidates for drug and Lf delivery since they are transported across cell membranes. When administered intravenously, liposomes naturally accumulate in the organs of the reticuloendothelial system, and they have been studied for drug delivery in cancer therapy (Mozafari, 2005; Mozafari et al., 2008). NLs have been utilized as drug carriers for a variety of substances, such as small-molecule drugs, proteins, nucleotides and plasmids, enhancing their activity by improving their stability and permeability and providing targeting and time release (Ishikado et al., 2005; Abu Lila and Ishida, 2017).

Ma et al. compared the effect of NLs containing Lf with that of natural Lf in a Caco-2 cell culture system. Lf-NL phospholipids were stable when evaluated using fatty acid peroxidation assays. The viability of colon cancer cells treated with Lf-NLs was substantially diminished, as determined by their metabolic activity and tests of cell numbers and proliferation. The effects of Lf-NLs on Caco-2 cells included a decrease in membrane integrity (lactate dehydrogenase leakage assay) and ROS generation. Cancer cells also showed morphological changes indicative of apoptosis after acridine orange/ethidium bromide (AO/EB) double staining. Thus, NLs containing Lf might be more efficient against CRC than natural glycoproteins (Ma et al., 2013).

Sugihara et al. evaluated the anti-inflammatory and antitumor effects of liposomal bLf (LbLf) on F344 rats treated with 1,2-dimethylhydrazine (DMH)/DSS. Bovine Lf was coated in soybean lecithin and exhibited improved stability in the stomach and increased absorption by the intestinal tract compared to bLf alone. DMH is a carcinogen that is widely used to study CRC since it induces the formation of ACF and is involved in the pathogenesis of this cancer. In the assay, rats were randomly divided into three groups: control (water) and groups treated with 500 or 1,000 mg/kg/day LbLf. The rats were injected with DMH (20 mg/kg) once per week for 8 consecutive weeks after 1 week of drinking water containing 1% DSS. All rats were sacrificed at 25 weeks. The tissues were examined for the presence of ACF and for a histopathological analysis. Additionally, human colon cancer cells were utilized to investigate the effect of LbLf on proliferation and inflammation. Rats from the 500 and 1,000 mg/kg/day LbLf groups showed significantly fewer colon ACF, adenomas, and adenocarcinomas than the rats from the control group. The authors also observed that LbLf inhibits cell growth and TNF-α mRNA expression. Therefore, LbLf affects CRC by suppressing inflammation and cell proliferation in rats. This *in vivo* study allows us to infer the preventive and therapeutic value of liposomal bLf in the treatment of human CRC (Sugihara et al., 2017).

### 4.2 Chitosan Nanoparticles Loaded or Coated With Lf

Chitosan is an N-deacetylated derivative of chitin that is present naturally and abundantly in crab and shrimp shells. Chitosan is a nontoxic, biocompatible, biodegradable, and adsorptive material. In addition, low-molecular-weight chitosan (LMWC) exerts a cytotoxic effect on oral cancer cells (Wimardhani et al., 2014). Chitosan NPs are a drug carrier with wide development potential and have the advantage of slow/controlled drug release, which improves drug solubility and stability, enhances efficacy, and reduces toxicity. In addition to the properties of chitosan *per se*, chitosan NPs can be modified and are muco-adhesive. Because of their small size, chitosan NPs are capable of passing through biological barriers *in vivo*. Therefore, chitosan NPs are used to improve the stability and efficacy of many drugs, including anticancer compounds (Wang et al., 2011). Abu-Serie et al. developed nanocombinations of Lf coated or loaded with lactoperoxidase (LPO, an enzyme present in milk showing antioxidant activity and the ability to degrade carcinogenic compounds). These LPO-loaded chitosan NPs with Lf exhibited increased stability and activity compared to single (free or nanoformulated) bovine proteins. The coating or loading of LPO-loaded NPs with Lf resulted in the highest synergistic cytotoxic effect on Caco-2 cells and greater selectivity in terms of the apoptosis-mediating anticancer effect than other NPs and the free proteins or 5-FU, without causing toxicity in normal cells. This synergistic increase in the anticancer activity was due to apoptosis, which was confirmed by substantial alterations in cellular morphology, a high percentage of annexin-stained cells and sub-G1 populations and nuclear staining with orange fluorescence in treated cancer cells. Additionally, significant alterations in the expression of well-characterized cellular proliferation and apoptosis markers (NF-κB, Bcl-2, and p53) were detected in NP-treated cancer cells compared to 5-FU-treated cells. Although these NPs have not been assayed in animals, they are promising reagents for the development of treatments for human CRC (Abu-Serie and El-Fakharany, 2017).

In another study, Kanwar et al. validated the efficacy of holo-bLf (Fe-bLf) in CRC by targeting survivin to kill colon cancer stem cells. The authors formulated nanocarriers/nanocapsules (NCs) using a complex preparation with calcium phosphate and chitosan and obtained a nanoformulation with a size of 200–250 nm. Fe-bLf was conjugated to the chitosan nanocores using carbodiimide-succinimide for the cross-linking reactions, and the nanocores were coated with alginate solution and calcium chloride (Kanwar et al., 2015). Survivin is a small protein that promotes cancer cell survival by inducing cell cycle progression and inhibiting cell death, suggesting that it may be a molecular target of cancer therapy (Yamamoto et al., 2008). The authors identified the roles of various miRNAs in absorption of the NCs/iron in various mouse organs and tissues. Interestingly, NCs reduced the viability of Caco-2 cells and cancer stem cell markers in triple-positive CD133, survivin and CD44 cancer stem-like cells. In addition, mice treated with NCs did not develop any tumors in a xenograft colon cancer model. One of the receptors for NC internalization is LfR, and in addition to inhibiting angiogenesis and the expression of stem cell markers, NCs also maintain iron and calcium levels (Kanwar et al., 2015).

Wang et al. performed an experiment in murine colon cancer cells (CT26) and human umbilical vein endothelial cells (HUVECs). In addition, they conducted *in vivo* experiments using the subcutaneous xenograft CRC model by performing s.c. injection of CT26 cells into the backs of Balb/c nude mice. The authors reprogrammed the tumor immune microenvironment (TIME) and metabolism via biomimetic targeted codelivery of the anticancer drugs shikonin (SHK) and JQ1. SHK is a phosphatase inhibitor that interferes with cellular signaling mediated by TNF-α and NF-κB (Kondapi, 2020), and JQ1 is a first-in-class potent and selective inhibitor of the Bromodomain-containing protein 4 (BRD4) signaling pathway; it is widely used for tumor biology studies (Shi et al., 2018). Mannopyranoside-Lf (Man-Lf) NPs were prepared using a green method of thermal denaturation, and anticancer drug-encapsulated Lf NPs were obtained using the same method; these NPs were i.v. injected in the animals. The use of Man-Lf-NPs is based on mannose receptors that are expressed on tumor-associated macrophages (TAMs) and facilitate the localization of the NPs and subsequent inhibition of lactate production (Kondapi, 2020). Interestingly, a confocal laser scanning microscopy examination of tumor sections showed that the Man-Lf NPs significantly increased calreticulin (CRT) expression on the membrane of tumor cells, the CRT protein is upregulated in cancer (Zamanian et al., 2013). In addition, significantly greater accumulation of Man-Lf NPs in tumors was observed than that of Lf NPs (Wang et al., 2011). The results suggest that Man-Lf-NPs could be used to target CRC cells in patients.

### 4.3 Use of Aptamers and Theranostics in CRC

Aptamers are short single-stranded DNA or RNA molecules that can be isolated from large combinatorial libraries through the systematic evolution of ligands by exponential enrichment (SELEX) procedure (Ellington and Szostak, 1990; Cui et al., 2016). They recognize target proteins, ranging from small molecules to proteins on whole cells, with high affinity and specificity; aptamers have similar properties to antibodies, although they are nanostructured molecules and easily internalized, in addition to showing thermostability, target adaptability, low immunogenicity, and resistance to denaturation (Zhong et al., 2020). All of these properties of aptamers make them potential diagnostic tools for clinical use, such as diagnosis and drug release. In addition, as cancer cells express various tumor-associated membrane proteins on their surface, aptamers combined with molecules recognizing these proteins can target the drug to the cancer cell (targeted therapy). A disadvantage is that aptamers are cleaved by nucleases, but this limitation is solved by modification of the molecule to protect it from these enzymes. Aptamers can be used in the diagnosis, prognosis, and therapy (theranosis) of cancer. Recently, a new technique of cell-based SELEX has been developed and used in CRC; this technique exhibits higher affinity and specificity than SELEX since the target cells are used to screen aptamers (Cui et al., 2016; Chen et al., 2017; Ahmadyousefi et al., 2019).

The first attempt to combine the anticancer therapeutic effects of bLf with the multimodal imaging efficacy of Fe_3_O_4_ NPs was reported by Roy et al. This combination (Fe_3_O_4_-bLf) was encapsulated in alginate-enclosed chitosan-coated calcium phosphate (AEC-CP) nanocarriers targeted with locked nucleic acid-modified aptamers against epithelial cell adhesion molecule (EpCAM) and nucleolin. This nanoformulation was orally administered to mice injected with triple-positive (EpCAM, CD133, and CD44) colon cancer stem cells in a xenograft cancer stem cell mouse model. The authors analyzed an ample list of apoptotic, stem cell and angiogenesis markers, cytokines, and gene expression of signaling molecules important in cancer. Interestingly, complete regression of tumors was observed in 70% of mice fed nontargeted (NT) NCs (control), with 30% mice showing tumor recurrence after 30 days. However, only 10% of mice fed targeted NCs showed tumor recurrence, indicating a significantly higher survival rate (Roy et al., 2015). Tumor necrosis factor (TNF)-related apoptosis-inducing ligand (TRAIL) is a member of the TNF superfamily that activates the apoptosis pathway by binding to its associated death receptors DR4 and DR5 (Yuan et al., 2018). The second mitochondria-derived activator of caspase (SMAC)/direct inhibitor of apoptosis protein (IAP)-binding protein with low pI (DIABLO) protein is an essential and endogenous antagonist of IAPs (Zhao et al., 2020). The authors found that the anticancer mechanism of the NCs was mediated by TRAIL, Fas, Fas-associated protein with death domain (FADD)-mediated phosphorylation of p53 to induce the activation of second mitochondria-derived activator of caspases (SMAC)/DIABLO (inhibiting survivin) and mitochondrial depolarization, leading to the release of cytochrome C. Apoptosis was induced by the inhibition of the Akt pathway and activation of cytokines released from monocytes/macrophages and DCs (Roy et al., 2015). On the other hand, the recurrence of tumors in NC-fed mice mainly occurred due to activation of alternative pathways, such as mitogen-activated protein kinase (MAPK), extracellular signal-regulated kinase (ERK) and Wnt signaling, leading to an increase in the expression of survivin and other antiapoptotic proteins. Thus, these NCs might be used to treat CRC in patients, and they have the special ability to target tumors, as observed using near-infrared (NIR) imaging, magnetic resonance imaging (MRI) and computerized tomographic (CT) techniques. In this case, real-time cancer therapeutic imaging leading to targeted colonic adenocarcinoma therapy would be feasible. Furthermore, these NCs maintained the immunomodulatory properties of bLf (Roy et al., 2015).

On the other hand, Kamaluparam et al. modified the preparation of NCs to combine the nanotheranostic approach of FebLf NCs and their innate anticancer activity with live *in vivo* imaging using near infrared fluorescence (NIRF) real-time live mouse imaging technology. The authors orally administered NCs to CIMP1+/CIMP2−/CIN + colonic adenocarcinoma tumor-bearing C57 Balb C nu/nu-nude mice. The NCs exhibited great *in vivo* antitumor effectiveness, leading to a reversion of xenograft tumor growth over 90 days, which was the experimental period. NIRF real-time imaging revealed the selective localization of the NCs at the tumor site and subsequent inhibition of tumor growth. *Ex vivo* NIRF imaging of mouse organs showed increased tumor uptake and biodistribution in vital organs, including the spleen, intestine, and kidney, and the histopathological analysis revealed the lack of toxicity of NCs toward mouse tissues. These results confirmed the biocompatible, multimodal anticancer activity of these novel FebLf NCs for real-time cancer therapeutic imaging, leading to targeted colonic adenocarcinoma therapy. Thus, FebLf NCs could be employed to treat human CRC in the near future (Kamalapuram et al., 2016). A review focused on the application of lactoferrin-loaded aptamers and anticancer drug delivery to solid tumors, specifically CRC that addressed the different targeted anticancer approaches was published by Chaudhary et al. (Chaudhary et al., 2017).

## 5 Conclusion and Perspectives

The intestinal inflammatory response is an orderly, controlled and organized process that facilitates tissue proliferation and repair. However, chronic inflammation may cause the loss of cellular homeostasis and trigger cellular alterations, including carcinogenesis. Bovine Lf and its peptides, either used alone or as adjuvants, are molecules that exert beneficial effects on inflammation and neoplastic cell proliferation in CRC, participating directly in carcinogenesis or in the modulation of the immune response to this cellular process. The dosage of administered bLf alone or in combination with chemotherapeutic drugs should be accurately measured to provide benefits while avoiding potential risks related to a proinflammatory environment that promotes tumor cell development in the intestine. Trials examining patients with CRC have documented the efficacy of orally administered bLf in the therapy course. Novel nanoformulations of bLf have been investigated in animal models with promising results. In addition, Bovine Lf can be used as a biomarker and a noninvasive diagnostic supporting test in patients with inflammatory disease or infectious bowel disease. Currently human recombinant lactoferrin (Kruzel et al., 2013) may provide advantages given its compatibility to be used for future in clinical trials to test its anticancer activity.
